# Fabrication of polyvinyl alcohol-graphene nanosheet nanocomposite loading of omega-3 fatty acids for ceramic engineering

**DOI:** 10.22038/IJBMS.2022.62366.13796

**Published:** 2022-03

**Authors:** Pegah Bitaraf, Azadeh Asefnejad, Nahid Hassanzadeh Nemati

**Affiliations:** 1 Biomedical Engineering Department, Islamic Azad University, Science and Research Branch, Tehran, Iran

**Keywords:** Graphene nanosheet, Neural tissue engineering, Omega-3 fatty acids, Polyvinyl alcohol, Treatment

## Abstract

**Objective(s):**

Many people all around the world encounter major problems due to nervous system injuries. Among the various methods of treating, neural tissue engineering has attracted a lot of attention from nerve science researchers.

**Materials and Methods:**

There are various methods for fabrication of soft tissue, however the electrospinning method (ELS) is a simple and cost-effective method that can produce porous fiber scaffolds to simulate the environment of the extracellular matrix (ECM). In this study, an ELS technique was used to fabricate polyvinyl alcohol (PVA) tissues and graphene nanosheet (Gr-NS) added with omega-3 fatty acids (O3FA) was loaded in these tissues that support nerve tissue regeneration. For this purpose, PVA and Gr-NS for biaxial ELS, PVA containing 0.5 wt%, and 1 wt% of Gr-NS was used.. Then, the morphology of these scaffolds was observed by optical microscopy and scanning electron microscopy (SEM) technique.

**Results:**

The results show after loading of O3FA, the fiber diameter reaches 0.573±0.12 µm, which is within the range of dimensions required for nerve tissue engineering. FTIR analysis indicates that Gr-NS and O3FA have been well loaded in the scaffolds. The results of water absorption and biodegradation tests demonstrated that the sample with 0.5% Gr-NS has 211.98% and 16.54% water absorption and biodegradation after 48 hr and 6 days, respectively.

**Conclusion:**

Finally, the results of this study indicate that scaffolds loaded with 0.5% Gr-NS have a homogeneous, porous, and integrated structure which can be effective in nerve tissue engineering.

## Introduction

In the peripheral nervous system (PNS), direct reconnection is the most common treatment for incisional nerve injuries in small incisions. Nerve autografts are the best choice for filling larger nerve gap problems. However, transplant deficiencies, potential loss of function in the transplant site, and the need for multiple surgeries are limiting reasons for autograft use (1-3). Allografts and xenografts are also intended to replace autologous nerve grafts. However, such treatments have been discontinued due to immune rejection and the possibility of disease transmission. Nerve repair in the central nervous system (CNS) is difficult, and an inhibitory environment is created in the CNS after the injury, which often limits nerve repair. Recent treatments are not sufficiently effective to keep nerve function in the CNS (4-7). For the above reasons, soft tissue engineering can be another option for implantation and to enhance nerve repair. The mechanical and chemical features of synthetic bonds may be adapted to nerve approaches. The biodegradable and polymer biocompatible substances are usually used to decrease the immune system responses (8-11). To control and monitor the morphology of the scaffold, fabrication and components are used to provide a suitable environment for tissue repair. These synthetic tissues may also be enhanced to provide an acceptable matrix for axons to penetrate the affected area in CNS soft tissue engineering. One of the common approaches in soft engineering is to imitate the natural ECM shape (12-15). The ECM plays a significant role in setting the cellular behaviors by affecting cells with biochemical, structural, and morphological signs. The ECM technology has two different components such as polysaccharides and fibrous proteins (16-22). 

Likewise, nanofiber microstructures are widely used as potential substrates for tissue engineering. It can be hypothesized that growth of the ECM provides a more beneficial condition for cellular functions, such as adhesion of the cells, migration in the cell wall, and differentiation. At the same time, nanofibers have a high surface-to-volume ratio (23-28). Gladman *et al*. (20) indicated that peripheral nerve damage in mice improved by increasing O3FA unsaturated fatty acid content. Due to the long recovery time after peripheral nerve injury, alternative therapies are needed to protect the nervous system from damage and improve repair. Omega-2 has been shown to have high potential in many neurological diseases and acute traumatic injuries. Their study aimed to investigate the potential for improving and protecting O3FA in peripheral neurological diseases. 

The study showed that compared with other WT samples, omega-3 (fat-1)-fed mice produced longer neurons with more branches. Researchers (23) examined the effect of O3FA on nerve damage. Their study aimed to consider whether increasing the amount of this substance in the tissue can prevent spinal cord injury (SCI). Their study was performed on mice and the results showed that after pressure injury to the SCI, omega-3-fed mice did not recover more than normal omega-6-fed mice. Also, fewer microglia and macrophages are activated, resulting in less inflammation. Oriented polylactic acid (PLA) nanofiber scaffolds coated with Gr-NS were used to improve cell function (24). The results showed that Gr-NS coated samples significantly improved cell proliferation compared with graphene-free samples. The scanning electron microscope (SEM) images taken from these nanofibers show that the first sample is electrospun PLA fibers and the other samples are coated with 0.5, 1.5, and 1.5 mg/ml Gr-NS. As mentioned earlier, polyvinyl alcohol (PVA) has attracted much attention in tissue engineering due to its hydrophilicity and biocompatibility properties. It was also pointed out that the nanofiber structures created by the electrospinning (ELS) method due to the fabrication of a porous fibrous substrate can effectively simulate the extracellular matrix (ECM) and thus accelerate the healing of damage. In this study, the ELS method has been used to produce PVA nanofibers. Graphene nanosheets have also been added to scaffolds to improve their mechanical properties and conductivity. In addition to the above, to facilitate repair and cell interactions, omega-3s are loaded into scaffolds, which are released by scaffold destruction. Omega-3s can be effective in treating nerve damage. After fabrication, these scaffolds are subjected to morphological, chemical, and cellular evaluations.

## Materials and Methods

***Fabrication of porous scaffold***

A list of all raw materials used in this study is given in Table 1. To determine the optimal percentage of ELS, different percentages of the solution are usually prepared and then ELS is done with the optimal sample. First, PVA is dissolved in deionized water in which 2 g of PVA is dissolved in 30 ml of deionized water at 100 °C (25). To fabricate a homogeneous solution, the solution is placed on the magnetic stirrer for 24 hr. After complete dissolution of the PVA in water, some of the solution is separated for ELS of a single PVA scaffold. Then, different amounts of 0.5 wt% and 1 wt% of Gr-NS are added to the rest of the solution which is placed in separate containers. The samples are sonicated for 20 min to disperse the nanoparticles well into the matrix and then placed on the magnetic stirrer for 24 hr. The solutions required for ELS are separated from PV0.5G and PV1G, and 1 μl of O3FA dissolved in 20 ml of ethanol is added to the remaining solutions and set on a magnetic stirrer for 12 hr to create a homogeneous solution. The solution is allowed to evaporate the ethanol. The PV1GO and PV0.5GO samples are created based on the amount of graphene nanosheet content. Also, a 0.5 wt% solution of nanographene in deionized water is prepared for ELS with two nozzles. For this purpose, 0.5 wt% of graphene nanosheets are dissolved in water and set in the ultrasonic bath for 20 min. The solution is then placed on a magnetic stirrer for 12 hr at 140 rpm to obtain a homogeneous sample. After preparing the desired solutions, the samples are transferred to glass containers for the ELS process. In the ELS method, the polymer should be dissolved in a solvent and then a high voltage is applied to the solution to form a unique nanofiber. Achieving a homogeneous microstructure with fibers of a similar size is one of the main challenges in creating fibrous substrates. In this study, the prepared solutions were transferred to a 10 ml syringe at each stage and placed in an ELS machine. Then, the ELS process was performed at room temperature with a flow rate of 0.5 ml/hr and a voltage of 15 kV applied. The distance from the nozzle to the collector is set at about 20 cm and the rotation speed of the collector is set at 600 rpm in case the ELS process is done for 1–4 hr, depending on the thickness of the prepared fiber with the size of 5 × 7 cm.

***Morphological analysis of the nerve fibers***

In order to study the morphology of the scaffolds, the light microscopy technique was used. Then, the scanning electron microscopy (SEM) (TESCAN VEGA Czech Republic) was used to evaluate the morphology of the electrospun samples with the size 1 × 1 cm coated with a gold layer. To increase the electrical conductivity of the samples, a thin layer of gold was coated and applied with different magnifications. The Emitech K450X device (UK) was used to cover an ultra-thin layer of gold on the samples and the images were taken using an accelerator voltage of 20 kV.

***Fiber diameter evaluation***

The fiber porosity was calculated using Image-J software and also to analyze the fiber dimension the mean standard deviation was performed for three times (SD±3).

***Fourier-transform infrared spectroscopy (FTIR) analysis***

FTIR used to study the chemical agents and bonds present in the samples in the range of 4000–400 cm^-1^. To examine the functional group of the tissue scaffolds, powdered samples were mixed with KBr in a ratio of 1: 100, and then the infrared spectrum was taken from samples. In order to investigate the surface, functional groups using the FTIR technique Ultra shield 400 MHZ (Bruker) device (Germany) in the range of 4000-400 cm^-1^ and a resolution of 2 cm^-1^ was used.

***Water absorption evaluation***

To evaluate the water absorption and swelling of electrospun substrates of the sample such as PVA0.5G, PVA0.5GO, PVA1G, and PVA1GO, the samples were first separated from aluminum foil, weighed and the dry weight of the samples was recorded (W0). Each sample was then immersed in a Falcon containing 20 ml of Phosphate Buffer Solution (PBS) at 37.5 °C (W). Their weight (fresh weight, W) was measured again and the absorption rate was obtained from Equation 1 (26). Each test was repeated 3 times and the average was reported as the water absorption.

Equation 1: Water absorption = [(W-W0)/W0] × 100% 

***Degradability evaluation***

A biodegradability test was used to evaluate the degradation rate of electrospun samples of PV0.5G, PV0.5GO, PV1G, and PV1GO. For this purpose, the samples were first weighed (initial weight, W0) and then placed in 20 ml of PBS for 12 days in a thermoset with a rotational speed of 50 rpm. After 1 and 3 days, the samples were removed from the falcon tube and their weight was recalculated (W) and then immersed in PBS solution. The degradability of scaffolds was obtained from Equation 2 (27). Each test was performed for 3 samples and the average was reported as the degradation rate.

Equation 2: Biodegradability= [(W-W0)/W0] × 100% 

***Electrical conductivity***

To evaluate the electrical conductivity of the fibers (σ, S/cm) was performed on PV1GO and PV0.5GO electrospun films using the standard 4-probe technique. The samples were placed in the probes and the corresponding current was calculated after the voltage was applied. Flow conduction is calculated using the following equation:

Equation 3: σ (S/cm) = (2.44×10/S) × (I/E) 

Where σ is the electrical conductivity, S is the surface thickness, I is the current flowing through the other probes, and E is the voltage drop across the internal probe. 

***Cytotoxicity evaluation***

To investigate the MTT assay and the toxicity of PV0.5G, PV0.5GO, PV1G, and PV1GO samples and their influence on the growth of cells and proliferation of cells, the extraction section was conducted according to ISO-10993-5 standard. First, a specific number of 1 × 10^4^ cells with 100 μl of culture medium were poured into each well of 96-well culture plate and then incubated at 37 °C for 24 hr to allow the cells to adhere to the bottom of the plate. 

A constant amount of culture medium (RPMI) was investigated for the control sample. After that in the time that the cells adhered, the culture medium was removed from the cells as much as possible and 90 μl of the sample extract along with 10 μl of Fetal bovine serum (FBS) was joined to the culture well and exposed to these extracts for another 24 hr. After 1 and 3 days, the culture medium was removed and 100 μl of MTT at a concentration of 0.5 mg/ml was poured into each well and incubated for 4 hr. After 4 hr, the solution was removed from the cells and isopropanol was poured to dissolve the purple crystals. To better dissolve the MTT precipitate, the plate was placed on a mixer for 15 min. Then, the amount of solution in isopropanol was measured using an Elisarider device at a wavelength of 545 nm.

## Results

The fiber feature enhances the release of biochemicals, oxidation rate, number of proteins, release of the drugs in the system, and nucleic acids, which may be transported through the porosity of the fibers (29-52). In addition, a large surface area increases the contact surface between cells and fibers, thus increasing the chemical adsorption of cells. Therefore, nanofiber scaffolds can be used effectively in soft tissue engineering (53-58). Among the various methods for the production of nanofibers, ELS is the easy, most cost-effective, and most common method with many advantages over other methods. For example, in this method, the morphology can be controlled and as a result, suitable mechanical properties are achieved. Also, by controlling the production parameters, nanofiber scaffolds with suitable porosity and homogeneous structure can be created (2, 59-64). As a result, in this study, this method has been used to make a polymer matrix with PVA. The PVA is a biocompatible, hydrophilic, inexpensive, non-toxic, and biodegradable polymer that is widely used in medical applications such as dialysis membranes, wound dressings, artificial skin, cardiovascular instruments, scaffolds (65-68). 

**Figure 1:**
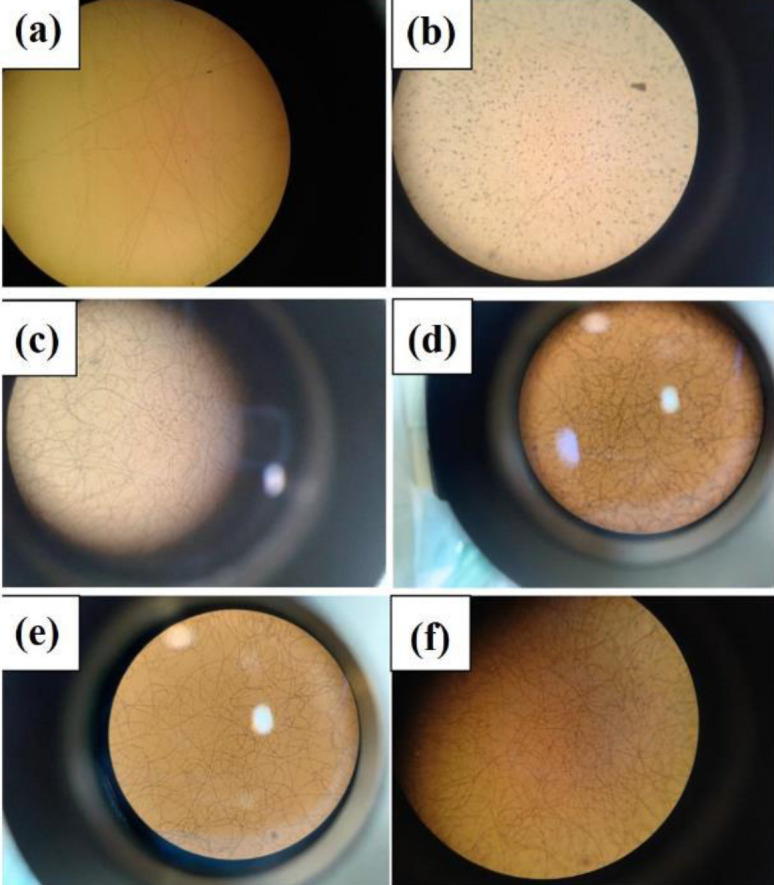
Electrified substrates a) PV, b) PVG2, c) PV1G, d) PV1GO, e) PV0.5G, and f) PV0.5GO on slide observed with a light microscope to examine fibers before ELS on aluminum foil

Graphene nanosheet is the thinnest two-dimensional (2D) nanomaterial in the world and has unique physicochemical properties. Graphene is used in a variety of fields such as antivirals, antibacterial, disease diagnosis, sensitive biosensors, cancer cell imaging, targeting and treatment, drug release, and tissue engineering. The use of graphene in nerve tissue engineering has recently been studied and welcomed by researchers (69-72). In fact, since nerve cells and their activities are dependent on electricity, the unique electrical properties of graphene can have many benefits in stimulating the nervous system and treating and diagnosing diseases. Graphene can also be functionalized by appropriate molecules to improve neural differentiation (5). As a result, graphene nanoparticles are loaded in these substrates to increase the mechanical properties, electrical conductivity, and function of nerve cells. Among the methods of making scaffolds, using ELS has attracted special attention in various studies (28). ELS also has the ability to create nano- and micro-fibers that can simulate the ECM (22). On the other hand, the porous structure is obtained and its porosity together leads to acceleration of water exchange, nutrients, and excretion of cellular waste, which is a significant and necessary thing in cell adhesion and growth (29). Also, because the surface rate is high compared with the volume of fibers produced in this method, cell-bed interaction can facilitate more. Hydrophilicity is one of the most important factors that affect cell adhesion, growth, and differentiation of the repair tissue (30). The PVA polymer, which is a very hydrophilic and biocompatible polymer was used to make scaffolds. The PVA has good ELS properties to make nanofiber with high tensile strength and good flexibility (31). In this study, the PVA substrate was first electrospun to create a free substrate with homogeneous fibers. The prepared PVA solution was then electrospun to create a suitable structure under a light electron microscope as shown in Figure 1a.

**Figure 2:**
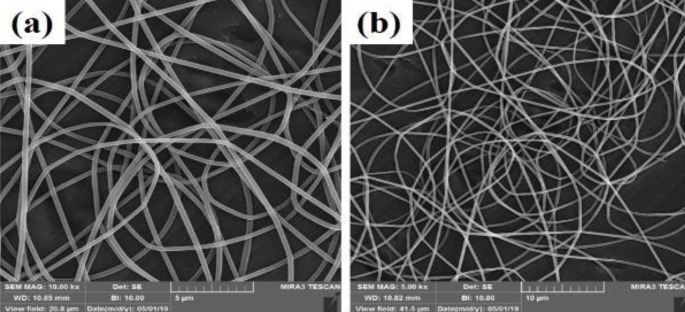
Scanning electron microscopy (SEM) images of polyvinyl (PV) fibers produced using ELS on aluminum foil containing various amounts of graphene nanosheet a) 500 nm and b) 400 nm

**Figure 3:**
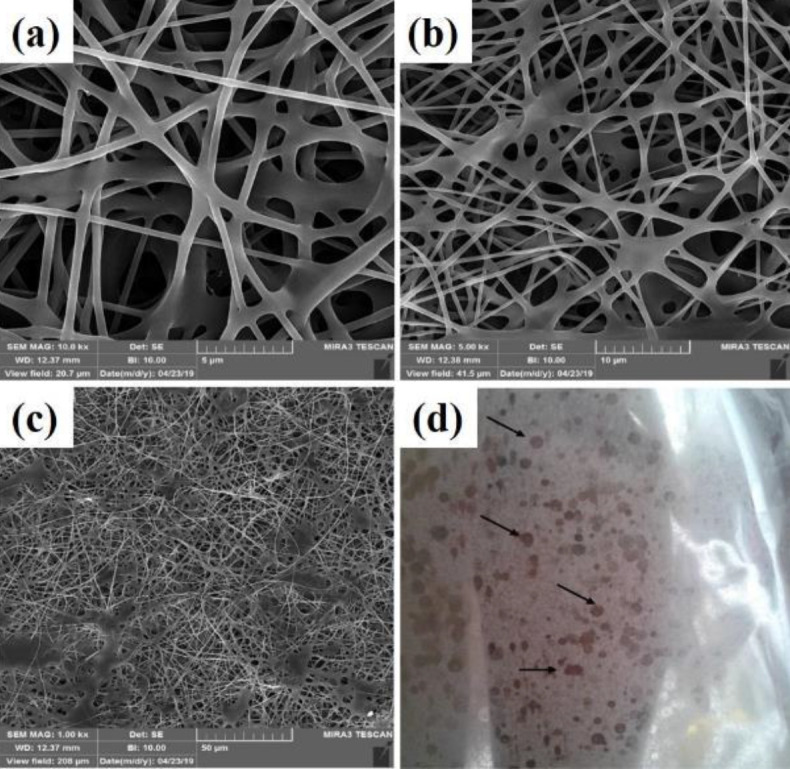
Scanning electron microscopy (SEM) images a-c) of PVG2 fibers and d) electrospun substrate PVG2

A homogeneous fibrous structure was created after the PVA substrate was electrospun on aluminum foil. The morphology of the scaffold created by SEM was observed in Figure 2 (a-b) with two magnifications. As can be seen, the structure of the created PVA is completely homogeneous and free of agglomeration. As the purpose of this study is nerve tissue engineering, graphene nanoparticles have been added to the ELS table. After confirming the homogeneity of the PVA matrix electrified by SEM tools, these substrates were electrified with two graphene nanoparticles as two nozzles. Observation of the light microscope of this substrate showed that the graphene nanosheet solution alone could not be electrospun. This can be attributed to the lack of proper viscosity. As shown in Figure 1 (b), graphene droplets are sprayed onto the slide under an electric field, which is not suitable for tissue engineering. The ELS bed of PVA and graphene solution is shown as two nozzles in Figure 3 (a-c). As can be seen, the graphene nanosheet has caused the PVA fibers to bond together and the fibers to be heterogeneous. Arrows indicate sprayed graphene droplets in which the substrate was not suitable for ELS and studies continued to achieve the optimal substrate. After the ELS Process performed, the nozzles sprayed composite containing graphene and PVA with a weight percentage of 1 wt% and added to the PVA solution.

**Figure 4:**
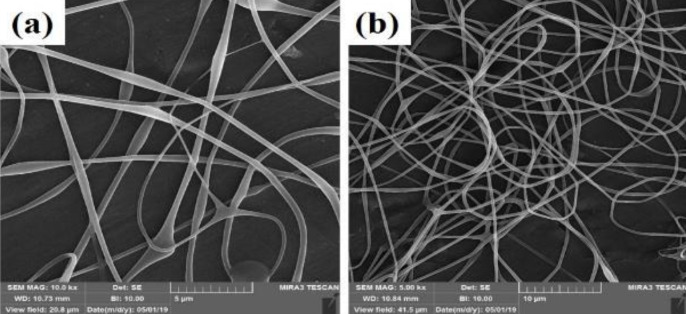
Scanning electron microscopy (SEM) images of PV1G fibers

**Figure 5:**
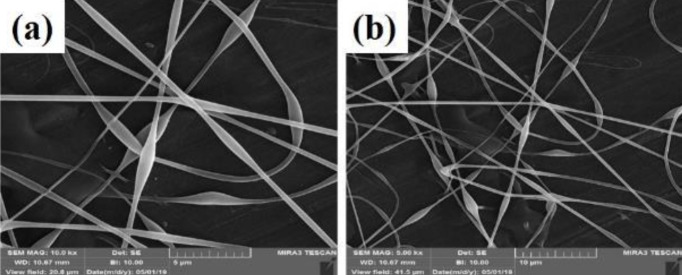
SEM images of PV1GO fibers

**Figure 6:**
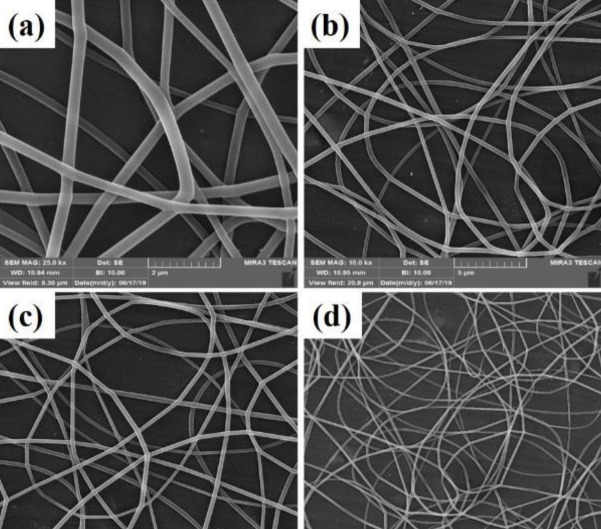
Scanning electron microscopy (SEM) images of PV0.5G fibers

**Figure 7:**
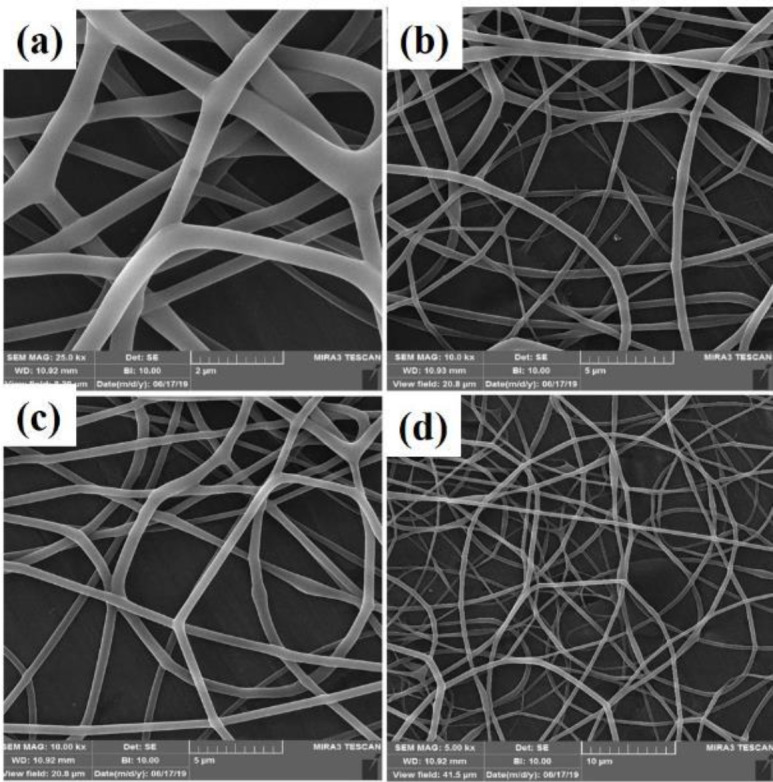
Scanning electron microscopy (SEM) images of PV0.5GO fibers

After ultrasonication and obtaining a homogeneous sample, ELS was performed on the slide, which is shown in Figure 4 (a-c). This amount leads to the formation of agglomerations in the spun fibers. The SEM images taken from this substrate also confirm this claim, which is shown in Figure 4 (a-b). After adding O3FA to this solution, the substrate was again seen under a light microscope, as shown in Figure 4 (d). The SEM image also confirmed the presence of agglomerations in the fibrous structure)Figure 5 (a-b). As a result, in order to reduce the viscosity and achieve a homogeneous porous fibrous substrate free of agglomeration, the weight percentage of graphene particles was reduced to 0.5 wt%. Then, the ELS process was performed on the slide. These fibrous substrates show that a homogeneous porous fibrous substrate was created without agglomeration. The SEM images of this substrate are shown in Figure 6 (a-d). This substrate maintains its homogeneous morphine-free morphology after addition of O3FAwhich as shown in Figure 1 (f) and Figure 7 (a-d). As a result, nutrients and oxygen are delivered to the cell and cellular wastes can be excreted, which is one of the important parameters for cell survival (34). The PV0.5G and PV0.5GO substrates are porous with interconnected porosity, thus providing a suitable space for the movement of water, nutrients, cellular waste, and cell growth. Taylor *et al.* (35) studied neurons that grew well on electrospun scaffolds because electrospun substrates produce well-connected porosity by simulating the matrix. ECM can provide a favorable environment for the adhesion and growth of nerve cells. As a result, PV0.5G and PV0.5GO samples were selected as optimal samples. Li *et al*., (36) use graphene as a substrate for nerve cells. According to the results of their study, graphene has high biocompatibility for neurons. It has also been shown that cell viability and mean neuronal length of graphene substrate are improved compared with polystyrene (36).

***Fiber diameter distribution***

Fibers have attracted special attention due to their long diameter at the nanoscale with many commercial and industrial applications. The properties of nanofibers that have attracted their attention include small diameter, high-level area, and low porosity size. These properties are very suitable for filtering, catalysis, adsorption, and tissue engineering (25-31). Mechanical reinforcement of fibers can be improved by adding nanofillers. Materials added to nanofibers as nanofillers include zero-dimensional nanoparticles, one-dimensional nanotubes, and two-dimensional laminates. Adding these materials can improve mechanical, electrical, thermal, or optical properties of the fibers. One of the most common two-dimensional nanofillers is graphene nanosheets, which have high mechanical, thermal, and electrical properties that improve the properties of fibers (22).

**Figure 8.**
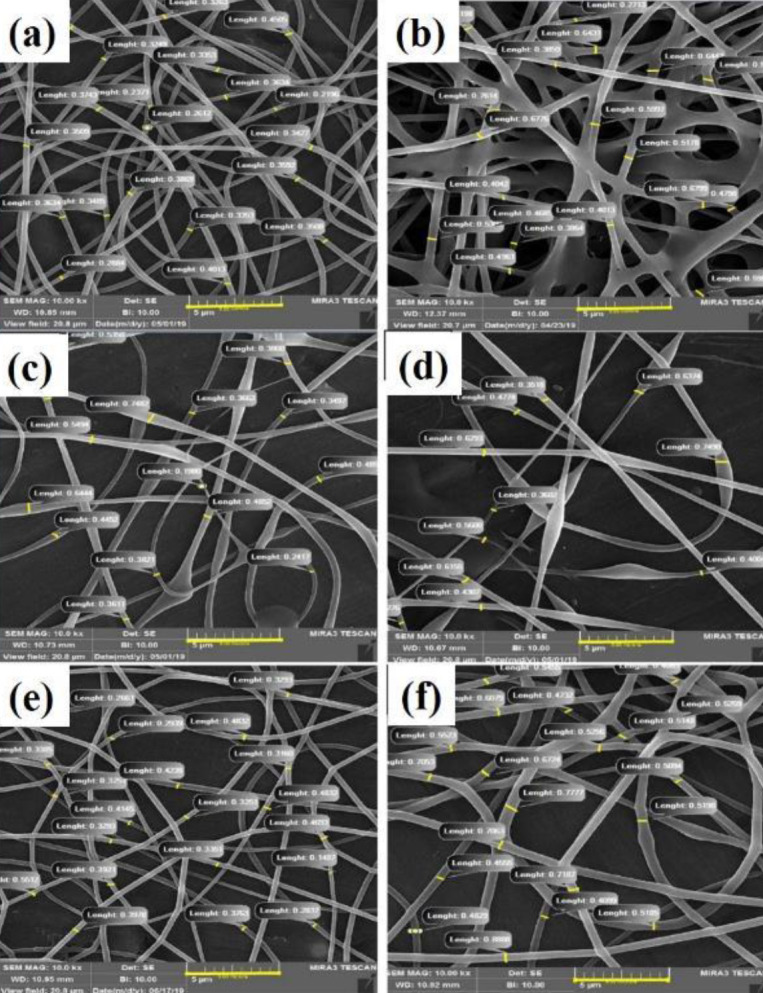
Measurement of fiber diameter on scaffolds a) PV, b) PVG2, c) PV1G, d) PV1GO, e) PV0.5G, and f) PV0.5GO by Image-J

**Figure 9:**
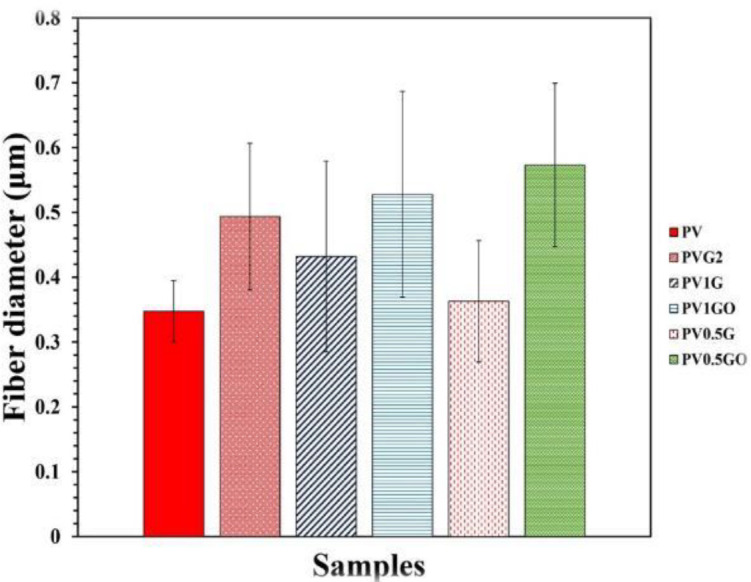
Fiber diameter diagram of PV, PVG2, PV1G, PV1GO, PV0.5G, and PV0.5GO samples

**Figure 10:**
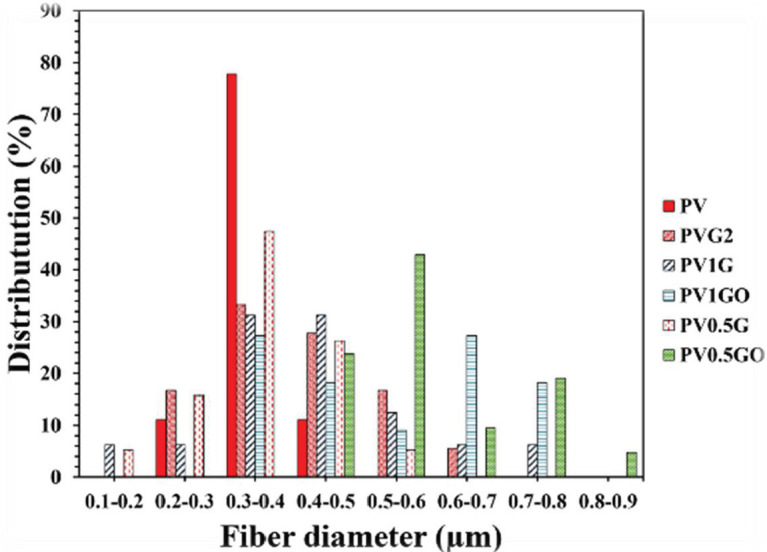
Fiber diameter distribution diagram of PV, PVG2, PV1G, PV1GO, PV0.5G, and PV0.5GO samples

In this study, measuring and examining the fiber diameter distribution of samples using SEM images using Image-J software has been determined and presented in Figure 8. Also, the results of measuring the diameter of fibers and their distribution are shown in Figures 9 and 10. According to the measurements, the fiber diameter sizes for electrospun scaffolds of 0.347±0.04, 0.493 ± 0.11, 0.431±0.14, 0.527± 0.25, 0.362± 0.09, and 0.573±0.12 μm were obtained for PV, PVG2, PV1G, PV1GO, PV0.5G, and PV0.5GO scaffolds. Although all the fibers obtained in the fiber range are suitable for nerve tissue engineering, the reasons for changing the diameter of the fibers and their distribution are discussed. As shown in Figure 9, the diameter of the fibers in the electrospun sample in the form of two nozzles (PVG2) is larger than in the pure PV sample. This can be due to the spraying of the solution and their adhesion due to the wetting of the pre-electrospun fibers. The obtained results of fiber diameter distribution of this bed show that this range of fibers is about 0.2 to 0.5 micrometers. The results also show that the size of PV1G scaffold fibers increased slightly in diameter compared with the pure PV sample, which can be attributed to the increase in solution viscosity. In the ELS process, increasing the viscosity leads to increasing the diameter of the fibers (36). Although the addition of graphene particles increases the conductivity, it also reduces the viscosity of the solution, which can lead to high thickness of the fibers and formation of agglomerationsA study (37) shows that increasing the content of polyethylene glycol in acidic polylactide scaffolds to more than 50% reduces the viscosity and as a result, an ELS structure is created. The decrease in viscosity is due to addition of low molecular weight polyethylene glycol content. Although this is less common in the PV0.5G sample, it may be due to the lower addition of graphene nanoparticles, which had a lower effect on viscosity. On the other hand, it is observed that the diameter of the fibers in the PV1G sample is scattered in terms of diameter, and no homogeneous scaffold is created. These fibers were in the dimension range of 0.8–1.8 micrometers. This can be due to formation of agglomerations and heterogeneity of fiber diameters. After reducing the number of nanoparticles to 0.5 wt%, i.e, in the PV0.5G sample, the range of fiber dispersion to 0.6–1.6 μm has been reduced, which indicates that the scaffold is more homogeneous. Also, in both PV1GO and PV0.5GO samples, the fiber diameter has increased compared with PV1G and PV0.5G samples, respectively. This can be attributed to the increase in viscosity and decrease in the conductivity of the solutions.

***FTIR analysis results***

**Figure 11:**
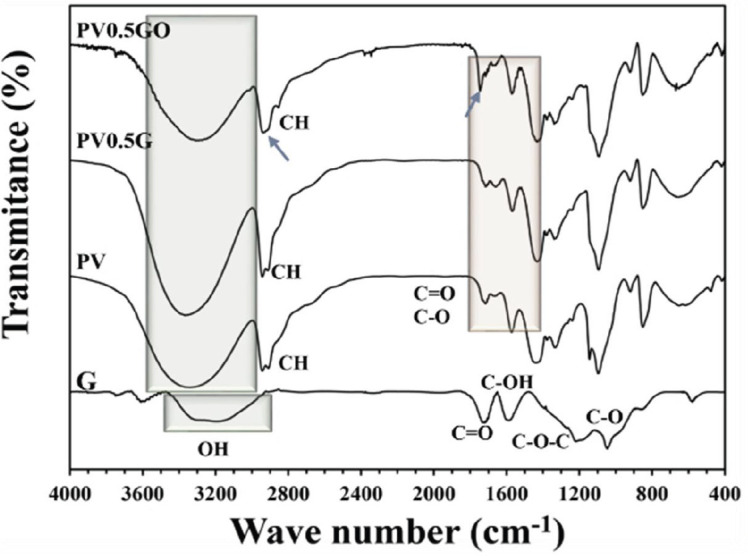
Infrared spectrum of G, PV, PV0.5G, and PV0.5GO samples

The raw materials were subjected to FTIR analysis to investigate the functional groups of raw materials and to ensure the loading of nanoparticles to the matrix. In the PVA spectrum, the broad peak between 3100–3600 cm^-1^ corresponds to hydroxyl. The peak seen between 2840 and 3000 cm^-1^ belongs to the CH tension and the peaks shown between 1680–1730 cm^-1^ correspond to C = O and CO of the remaining acetate groups in PVA, which are shown in Figure 11 (38). In the graphene nanosheet spectrum, the peak seen at 1066 cm^-1^ is related to C-O traction. The peak observed at 1288 cm^-1^ also confirms the C-O-C bending. C-OH bending is also seen at 1587 cm^-1^. The wide peak seen from 2950 to 3300 cm^-1^ can be attributed to the tensile vibration of the hydroxyl group. The C = O tensile peak is also observed at 1724 cm^-1^ (39). The peak reduction observed at 1150 cm^-1^ in PV0.5G and PV0.5GO samples is due to the interaction of PVA and graphene (25). The peak shown in the PV0.5G and PV0.5GO diagrams between 3000 and 3500 cm^-1^ is related to OH tension, which indicates a strong intermolecular and intramolecular hydrogen bond. However, in these two diagrams, the peak is shifted to the right relative to the pure PVA sample, indicating H interactions. On the other hand, in the PV0.5GO diagram, the peak C = O is shifted slightly to the left, which indicates the hydrogen interaction between O3FA and the structure of PVA and graphene nanosheet. Peaks seen between 2840 and 3000 cm^-1^ belonging to C-H traction are re-observed in both samples (40). The peak observed at 2923 cm^-1^ of the PV0.5GO sample is related to the asymmetric traction of CH_2_ in O3FA. Also, the peak appearing at 2852 cm^-1^ belongs to the simultaneous tension of CH_2_. Also, the amplified peak of C = O at 1730 cm^-1^ is related to the ethyl ester form of O3FA (41).

***Water absorption***

**Figure 12:**
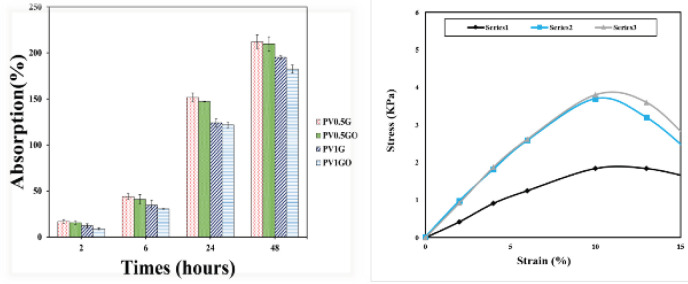
Water absorption (%) of electrospun substrates PV1G, PV1GO, PV0.5G, and PV0.5GO after 2, 6, 24, and 48 hr of immersion in PBS solution

Water uptake and swelling are important parameters in the transport of water, food, and waste products due to metabolism in applications such as tissue engineering, nerve repair, and drug release (29-32). Also, the ability to absorb water leads to an increase in the percentage of porosity and the size of the porosity, when the scaffolds are placed in a humid environment, and the penetration of cells into the structure, resulting in three-dimensional growth (42-43). One of the factors affecting the percentage of water absorption is porosity and its coexistence. The interconnected porosity obtained by the ELS method allows the rapid absorption of water to the center of the bed with the support of penetration and capillary force and thus increases the rate of water absorption (44-49). The water absorption capacity of electrospun substrates PV1G, PV1GO, PV0.5G, and PV0.5GO in this study was calculated using immersion in PBS solution. The water absorption results of these samples after 2, 6, 24, and 48 hr of immersion in PBS solution are shown in Figure 12. All of these electrospun substrates show good water uptake, which can be attributed to the hydrophilicity of the materials used, as well as the porous structure of the substrate and the interconnection of the pores. The P0.5G sample was about 16.85%, 43.91%, 151.53%, and 219.98% water absorption after 2, 6, 24, and 48 hr of immersion in PBS solution, respectively. The PVA is a hydrophilic polymer due to the numerous hydroxyl groups in its structure (45). On the other hand, comparing the PV1G sample with the PV0.5G sample, it is observed that after loading graphene nanoparticles in the structure, the water absorption of the sample decreased. The use of graphene nanosheet along with PVA leads to physical crosslinking. The hydrogen of the OH and carboxyl groups of graphene react by the atoms of the oxygen and the OH groups in PVA. On the other hand, H atoms of OH groups in PVA may have a chemical reaction with oxygen atoms of OH, carboxyl, and carbonyl graphene nanosheet groups (46). The higher the number of cross-links in the sample, the harder it is for water particles to pass through the structure, resulting in reduced water absorption. The plate nature of graphene also makes it difficult for water to pass through (47). It is also observed that samples loaded with O3FA have less water absorption compared with those without O3FA. In fact, the amount of this reduction is very small, which is due to the low amount of O3FA. Although O3FA are both hydrophilic and hydrophobic, their nature is more hydrophobic (48) but the presence of this substance can fill more voids and make water more difficult to pass. This could be another reason for less water absorption of samples with more graphene particles and more O3FA.

***Weight loss and weigh degradability***

**Figure 13:**
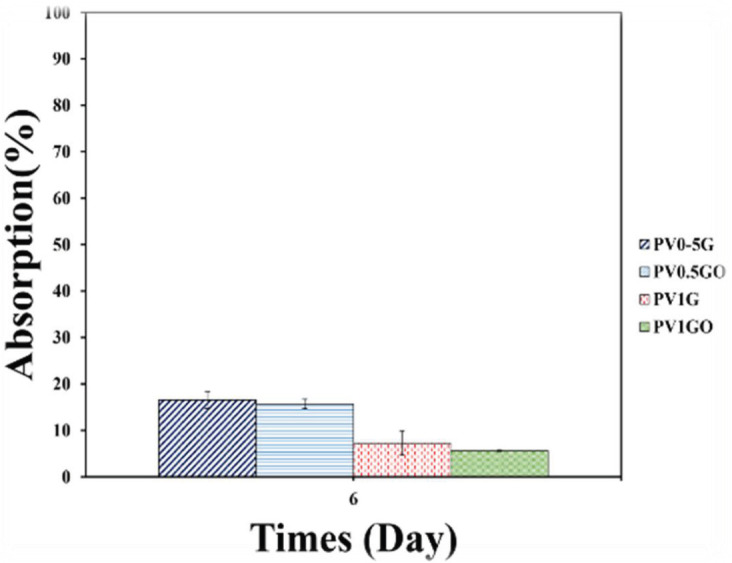
Degradation of electrospun substrates PV1G, PV1GO, PV0.5G, and PV0.5GO after 6 days of immersion in PBS solution

Tissue engineering scaffolds must be degradable to provide sufficient space for cells to grow and new tissue to form (49). Therefore, the tissue engineering structure must be designed to be destroyed at the appropriate time. Although scaffold degradation rate is an important factor, balancing scaffold degradation with tissue formation is not easily achievable because of various parameters such as site size and shape, and release of acidic products from scaffold degradation that can lead to responses (50). The amount of hydrophilicity is one of the most important factors affecting the rate of destruction of scaffolds (51). More hydrophilicity accelerates the degradation process (52). PVA is a hydrophilic polymer and its degradation occurs rapidly. The type of selected materials, hydrophilicity, type and percentage of crosslinker used and structure porosity are the factors affecting the rate of scaffold destruction. The porosities produced are connected by the ELS method and provide a suitable space for the exchange of water and liquids, which affect the water absorption and degradation rate of scaffolds and accelerate it. In this study, the extent of scaffold degradation was investigated by immersion in PBS solution for 6 days as shown in Figure 13. The results show that the PV0.5G sample had 16.54% degradation after 6 days, which is more than other samples. The obtained results for degradation are consistent with the results obtained from water uptake. In this test, degradation decreases with increasing graphene or O3FA. This may be due to lower hydrophilicity of these samples. It can be said that graphene nanoparticles act as physical cross-linkers and the presence of graphene nanoparticles acts as a barrier to the passage of water. Adding crosslinks can reduce the rate of bed degradation. In the PV1G sample, the degradation decreased to 7.23% after 6 days. (53). A study (54) showed that the higher the percentage of crosslinkers used, the lower the rate of inflation and consequent degradation.

***Electrical conductivity***

**Figure 14:**
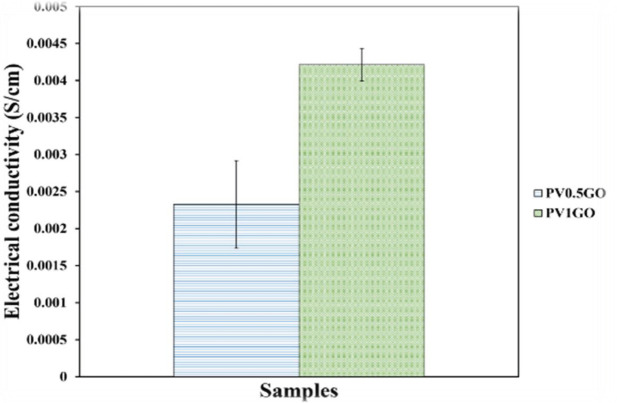
Guidance of PV0.5GO and PV1GO samples

Neuron growth and migration of the cell are essential issues for the nerve repair system. Axon-guided expansion toward targets to modify synaptic connections may support improving neural function. Glial cells migrating to damaged sites may secrete neurotrophic parameters and use repaired neurons. Accordingly, the ability to increase neuronal growth, direct axon expansion, and facilitate cell migration is an important factor in the preparation of soft tissue for the nerve repair process. The micro and nano matrices have been extensively considered to understand the influence of contact conduction on the behavior and function of neurons (1). Low conductivity of polymers limits their use to the extent to which electrical stimulation is required for neural tissue engineering. Electrical conduction has been reported to increase neurons, expand axons, and accelerate the nerve repair process. Therefore, research has led to the loading of a conducting particle into a polymer matrix to create a substrate with suitable properties (32). On the other hand, previous studies have shown that graphene nanosheets have many benefits, including good electrical conductivity, biocompatibility, high electrical stability, and good mechanical properties (32-38, 70). Addition of graphene nanosheet increases the mechanical properties of the polymer matrix (33). In this study, graphene nanosheets were loaded to increase the conductivity of the PV0.5GO and PV1GO samples using a 4-probe test as shown in Figure 14. As already known, the amount of electrical conductivity has increased with increasing percentage of graphene nanosheet and this amount can be effective in the growth of neuronal cells.

***Cellular studies***

**Figure 15:**
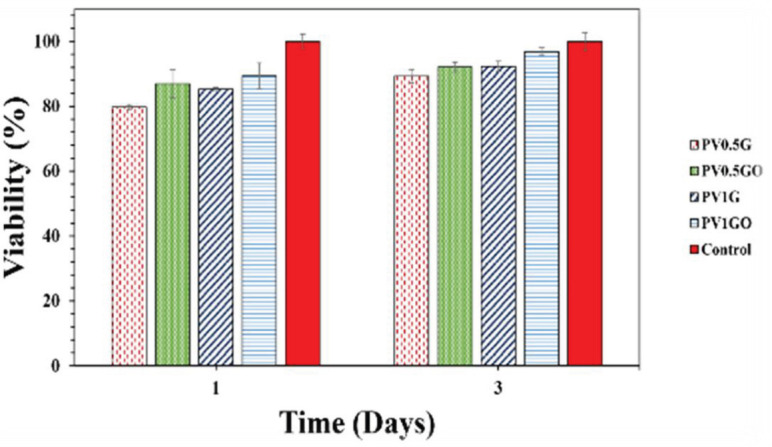
Cell viability after 1 and 3 days of exposure to electrospun scaffold PV1G, PV1GO, PV0.5G, and PV0.5GO by MTT assay

The ability of biomaterial substrates to support cell adhesion and cell proliferation is a very important factor (55-56). Among the various scaffold methods, electrospun scaffolds simulate the structure of the ECM of natural tissue, which leads to improved adhesion and cell growth (57). In this study, cell survival after 1 and 3 days of exposure to electrospun scaffold PV1G, PV1GO, PV0.5G, and PV0.5GO was determined by MTT assay as shown in Figure 15. The electrospun PVA layer and graphene nanosheet can physically simulate the structure of an ECM composed of random nanometer-sized fibers. As a result, the cells showed good cell viability with highly biocompatible properties. Hydrophilicity of porous scaffold is one of the key requirements in cell adhesion and cell proliferation. In fact, the porous scaffold hydrophilicity is effective on protein adhesion and cell adhesion (59). Neurotrophic factors have profound effects on nerve growth and function. These factors are usually expressed in large amounts in the presence of nerve damage and play an important role in supporting the survival of nerve cells and axonal regrowth. Numerous transmission methods for the entry of these factors into damaged sites have been extensively studied in both CNS and PNS systems, including in the form of microspheres and polymer matrices. Electrospun nanofibers may be a promising means of transmission because of their simultaneous ability to provide scaffold function and contact conduction, which may be required for nerve repair.

**Table 1:** Materials used in the study for preparation of nanocomposites

*Properties*	Chemical formula	Materials
*Molecular mass = 72000*	(C_2_H_4_O)x	Polyvinyl alcohol
*Derived from fish oil*	C_60_H_92_O_6_	Omega 3
*Molecular mass = 48/4239 g/mol*	--	Nanographene sheet
*15-20 nm*	C_2_H_5_OH	Ethanol
*--*	H_2_O	Deionized water

## Discussion

The porous nanofiber structure also facilitates the exchange of oxygen, water, and nutrients which allows the cell to penetrate the three-dimensional structure (58). However, it has been observed that with an increase of graphene nanosheet as well as O3FA, a very small amount 

of biocompatibility increases, despite the decrease in hydrophilicity (59-72). This may be due to the higher bioactivity provided by graphene nanosheets and the release of O3FA to support cell growth. The results of this study are consistent with the study conducted by other researchers (60) showing that with an increasing graphene nanosheet, the biocompatibility of chitosan/PVA scaffolds increased. This change has been attributed to the higher biocompatibility and bioactivity of graphene nanosheets. Further studies are required to confirm the features of porous scaffolds (61-69).

Nerve tissue regeneration requires a scaffold that should ideally be biodegradable, biocompatible, and have mechanical strength, large area, high porosity, and interconnected pores, as well as a bridge for axonal regeneration. The high porosity of the nanofiber scaffold gives more structural space for the cells to be located, enabling the exchange of nutrients and metabolic wastes between the scaffold and the environment. Electrospinning is one of the new techniques in the production of nanofiber scaffolds that mimic the functional morphology and structure of the extracellular matrix (ECM) and means the use of very high voltages to produce nanofibers. Adding these materials can improve the mechanical, electrical, thermal, or optical properties of the fibers. One of the most common two-dimensional nanofillers is graphene nanosheets, which have high mechanical, thermal, and electrical properties that improve the properties of fibers. Among the various methods for the production of nanofibers, electrospinning is the easiest, most cost-effective, and most common method. Electrospinning has many advantages over other methods. For example, in this method, the morphology can be controlled and as a result, suitable mechanical properties are created as shown in Figure 12 which shows that addition of graphene nanosheet has an upward trend compared with the other curve. Also, by controlling the production parameters, nanofiber scaffolds with suitable porosity and homogeneous structure can be created. As a result, in this study, this method has been used to make polymer substrates. PVA is a biocompatible, hydrophilic, inexpensive, non-toxic, and biodegradable polymer that is widely used in medical applications such as dialysis membranes, wound dressings, artificial skin, cardiovascular (CVD) instruments, and scaffolds. 

## Conclusion

Among various methods for fabrication of scaffolds in tissue engineering, the electrospun technique has been used to produce porous nanofiber scaffolds. For this purpose, porous scaffolds made of PVA, PVA, and graphene nanosheet were electrospun in the form of two nozzles in which PVA contained 0.5 wt% and 1 wt% of graphene nanosheet containing or without O3FA. First, before complete ELS of the scaffolds, the morphology of each substrate was observed on a slide under a light microscope to ensure their electrospun ability. Then, the morphology of this scaffold was observed by SEM tools. The obtained results show that scaffolds containing 0.5 wt% of graphene nanosheet were homogeneous and free of agglomeration and 1 wt% of graphene nanosheet leads to the agglomeration event. In addition, ELS of graphene nanosheet and PVA was observed to produce heterogeneous scaffold architecture. Then, the diameter of the fibers and the distribution of electrospun substrates were measured with Image-J software, which shows that after O3FA loading, the diameter of the fibers reaches 0.573±0.12 μm, which is in the dimensional range required for nerve tissue engineering. It was also observed that with increasing O3FA content, the diameter of the fibers increases, which is due to the increased viscosity and reduced conductivity of the solution. From the raw materials and substrates containing 0.5 wt% of graphene nanosheet, FTIR analysis indicated the nanosheet and O3FA have been loaded well in the scaffolds structure. Water uptake of samples by immersion in PBS solution was investigated for 48 hr. Examination of water absorption of samples and degradation showed that the sample had 0.5 wt% of graphene nanosheet, 211.98% water absorption after 48 hr of immersion, and 16.54% degradation after 6 days compared with other samples. The electrical conductivity test on the samples showed that these samples had proper electrical conductivity. The evaluation of cell survival indicated good adhesion and cell growth on the samples. Finally, the obtained results indicate that loaded scaffolds with 0.5 wt% of graphene nanosheet and containing O3FA had a homogeneous, porous, and integrated structure that is used in nerve tissue engineering. 

## Authors’ Contributions

PB was a Master’s student, AA Supervised, and NHN was a second supervisor.

## Conflicts of Interest

The authors declare that no conflict of interest exists.
